# Quality of Life of Elementary School Students with Type 1 Diabetes in a Developing Country during the COVID Pandemic

**DOI:** 10.3390/ijerph192214873

**Published:** 2022-11-11

**Authors:** Maja Raicevic, Aleksandar Obradovic, Mira Samardzic, Marija Raicevic, Natasa Curovic Popovic, Sanja Panic Zaric

**Affiliations:** 1 Institute for Children’s Diseases, Clinical Centre of Montenegro, 81000 Podgorica, Montenegro; 2 Institute of Public Health of Montenegro, 81000 Podgorica, Montenegro; 3 Faculty of Medicine, University of Montenegro, 81000 Podgorica, Montenegro; 4 Mother and Child Health Care Institute of Serbia “Dr Vukan Cupic”, 11000 Belgrade, Serbia

**Keywords:** diabetes mellitus, T1D, quality of life, HRQoL, COVID, students

## Abstract

Type 1 diabetes (T1D) is a condition that affects all aspects of life, and thus is closely related to the quality of life itself. Dealing with it during the COVID-19 pandemic is a big challenge. A case–control study conducted in Montenegro at the end of 2021 included 87 elementary school students with T1D and 248 of their peers as controls matched by gender. Standardized questionnaires were distributed to participants (Peds-QL Generic core 4.0 questionnaire for all participants and Peds-QL Diabetes Module 3.2 only for cases). Based on them, the results of obtained scores were measured and compared using non-parametric statistical methods in relation to gender, region and type of household. Children with T1D reported lower quality of life comparing to matching controls with lower scores in almost all domains. Differences in the same domains among patients and their classmates were also observed in the different gender subgroups, environment type subgroups and in the central region. Results of the study provide insights to prioritizing actions for children with diabetes care as well as for public healthcare planning.

## 1. Introduction

After being diagnosed with type 1 diabetes (T1D) children challenge many difficulties; they have to make corrections in food intake and physical activity, repeatedly check blood glucose during the day and night, get multiple insulin injections, all to target optimal glycemic range and best possible quality of life (QoL). 

QoL is acknowledged as an important indicator of diabetes care outcome, but also as an indicator of public health functioning [1,2]. It is influenced by many health-related, environmental and social factors. 

Children with type 1 diabetes, in comparison to their healthy peers, are challenged more frequently with depressive and anxiety disorders, which can be insulin-induced and lead to their poor metabolic control and lower quality of life [3,4].

Many research papers suggested that since the SARS-CoV-2 virus has been spread throughout the world, the COVID-19 pandemic significantly negatively impact the health-related quality of life (HRQoL) of children and adolescents [5,6]. It could be due to school closures, social distancing, changes in family environment, sedentary lifestyle, etc [7]. It was previously reported that during COVID-19 pandemic children and adolescents were frequently coping with mental disturbances such as anxiety, depression, stress, loneliness and tension [8].

There are scarce data on the impact of the COVID-19 pandemic on QoL of children with chronic diseases, such as T1D, although awareness about the risk of their disease during COVID-19 pandemic was raised. It is observed that patients with diabetes are in higher risk of severe presentation of COVID-19, the need for mechanical ventilation and mortality [9].

The aim of this study is to evaluate the QoL of elementary school students in Montenegro with T1D and to compare it with QoL of their peers, during the COVID-19 pandemic. 

## 2. Materials and Methods

During November and December 2021, the medical records of all patients diagnosed under the age of 15 with new-onset T1D, between January 1992 and April 2021 in Montenegro, was evaluated. 135 of them were elementary school students at the moment of study, aged 5–15. This study involved 90 of them who had regular check-ups in the endocrinology department of the Institute for Children’s Diseases, Clinical Center of Montenegro (where they have appointments every 3–4 months) as cases. They were offered the QoL questionnaires. Three families disagreed to participate in the study (two because of feeling unpleasant to participate and the third one because of being unlettered). A total of 335 elementary school students participated in the study, 87 (26.0%) were cases. A control group was formed of patients` classmates, without diabetes, who were matched by gender with a case–control ratio 1:3. After parent or caregiver signed the consent for participation, questionnaires were distributed to the patients’ school for the control group. To reduce bias, controls were elected as the first three of the same gender as their classmate with T1D, encountered in Teacher Diary among ordinal numbers 5 and 15. All participants completed the questionnaires independently. 

Participants were divided into groups regarding their place of residence, based on the type of the surrounding (rural/urban) and geographical region. It is an important point of this research, because of the availability of healthcare resources which are lacking in rural areas, especially in the northern part of the country. Differences among regions concern economic resources and life standard, which is highest in the south region and lowest in the north region of Montenegro.

The approvals of the Ethical committee of Clinical Center of Montenegro, Ministry of Education of Montenegro and Bureau for Education of Montenegro were obtained. 

The measurement tool was a standardized Peds-QL Generic core 4.0 questionnaire, covering 4 domains: physical health (8 questions), emotional health (5 questions), social functioning (5 questions) and school functioning (5 questions) [10]. Peds-QL Diabetes Module 3.2 is a diabetes-specific pediatric questionnaire authored by Varni et al. and for the purpose of this research, the Montenegrin language version was validated by MAPI Research Trust. It was filled up only by patients with T1D and it covers 5 domains: diabetes (15 questions), barriers to therapy (5 questions), adherence to therapy (6 questions), worry (2 or 3 questions/depending on age), and communication (4 questions) [11]. Each question is graded 0–4 (0-never, 1-almost never, 2-sometimes, 3-often and 4-almost always), then points are added to these values: 0 = 100, 1 = 75, 2 = 50, 3 = 25, 4 = 0. At the end, all points are added together, so the higher score the better the quality of life. Both, children with diabetes and control group filled out the Questionnaire for general data (age, place of residence, education, employment of the parents and their marital status). Data about metabolic control and insulin therapy were taken from the patient’s medical records.

EZR (Easy R) plugin (version 1.42) on R Commander (version 2.6–2) was used for descriptive statistics of the collected data and for analytic statistical data processing, to compare patients’ with control group reports, primarily for comparing medians with Mann–Whitney U test (due to data lack of normal distribution) and calculating the correlation coefficient (Spearman). The selected significance level was *p* < 0.05. 

## 3. Results

The control group was smaller (248) than planned (87 × 3 = 261) because in some rural areas were not enough students in the same class or even grade. Median of age in years was 12.4. Among participants 61.2% were boys. In the central region of Montenegro lived 48.1%, predominantly in urban surroundings (77.3%) (Table 1).

Children with T1D reported lower HRQOL than their matching controls (Table 2). They had significantly lower scores in domain of emotional and school functioning, as well as lower Psychosocial Health Summary Score. Differences in the same domains among patients and their classmates were also observed in the different gender subgroups, environment type subgroups and in the central region. Controls living in the north and south region of the country did not have better emotional functioning comparing to their peers with diabetes. 

The lowest median score was observed in domain of school functioning, in rural area, as well among boys with T1D and in the central region (Table 2).

Regardless the diabetes status, with older age “physical functioning” score slumps (Table 3). This moderate negative correlation is found among girls. It is also present in urban surroundings, but as a weak (Table 3).

According to the children with T1D reports and results of Peds-QL Diabetes Module, their median diabetes-related QoL score was 73.9 (IQR 64.4–82.0). The most important difficulties for patients concerned “worry” (median 58.3, IQR 50.0–75.0), followed by “barriers to therapy” (median 70.0, IQR 60.0–85.0). For only 33.7% has never been hard doing everything they need to care for diabetes, as they reported for the month before the research interview. Almost every third patient (33.7%) also reported, that at least once felt embarrassed about having diabetes during the same period.

Following, only 34.9% of patients reported that never felt weak, 54.7% reported that never had headaches, and majority of them reported feeling hungry, thirsty, “low” or “high” from time to time (Table 4).

## 4. Discussion

The results of the present study estimated that children with T1D in Montenegro have lower QoL in comparison to their non-diabetic peers. It is not a novel finding [12,13,14]. A similar research was conducted in Montenegro almost a decade ago, and students with T1D had also lower score in domain of school functioning in comparison to control group, but we have furthermore registered significant differences in their emotional functioning and psychosocial health [13]. 

The results of our study are consistent with previous reported from the Bekele et al. They have interviewed 379 patients with T1D, 5–18 years old, few months prior than our study was conducted. Their results revealed likewise lower scores in children emotional and school functioning, but adequate social functioning [15]. The highest scores in domain of social functioning in our study could be due to returning to school after COVID-19 lockdown and online teaching, mentioning our study was conducted between two peaks of COVID-19 pandemic in Montenegro in a relatively “stable” epidemiological period and just before the highest number at the end of 2021 and at the beginning of 2022 [16].

Low emotional scores could be the consequence of coping with puberty, trying to become independent and manage their disease, and further being preoccupied with chronic complications of diabetes [12]. Low diabetes-related QoL is probably attributed to demands to maintain optimal metabolic control. 

Remarkably low score in the section “worries”, showed that elementary school students with T1D, although very young, are deeply concerned regarding their health. They have problem in school functioning, especially if they live in rural area. All of the above emphasizes the need for psychological support.

Actually, it is already known that children with T1D have significantly impaired school functioning in comparison to their peers, but we have identified that problem is larger in the central region of Montenegro and for children living in rural surroundings [12,14,17].

Furthermore, some actions are needed on raising awareness about diabetes among school staff and patients’ peers, especially in central region of Montenegro and for those living in rural settings, so children with T1D would have positive self-concept. It could be diabetes training organized through workshops for staff and patients’ peers with an aim to fortify their relationship with patients and their parents. During COVID-19 pandemic an online education improved patients’ quality of life, which should be scrutinized as an important action in potential future crises [18].

Contrasting previously reported, our cases did not have more problems with physical functioning. Anyway, it is observed in our study that during COVID-19 pandemic, age have correlated with physical health in girls, which suggests that more attention should be paid to female students of higher grades.

The introduction of modern technologies in diabetes care, significantly improved metabolic control and QoL in patients with T1D, and it can concurrently reduce complaints about feeling “low” or “high”, but it must become available even for patients in developing countries [19,20].

Almost two-thirds of cases in our study reported feeling weak during the month before the interview, but it is not clear if it is the consequence of their chronic disease or it is just a COVID-19 infection symptom, because of unknown COVID status. 

One of the rare studies on QoL of children with T1D during COVID-19 pandemic-related lockdown, showed no significant differences in QoL in children and their parents reports comparing periods before and immediately after lockdown [21]. On the other hand, the adults with T1D, 18 months after SARS-CoV2 outbreak, reported worsened lifestyles; gaining weight and worsening quality of sleep [22]. Moreover, Welling et al. investigated the impact of COVID pandemic-related lockdown on eating behaviors, physical activity and QoL in children with severe obesity, and also observed deterioration [23].

Our study has few limitations. According to the lack of QoL assessment just before COVID-19 pandemic in our research, we are unable to appropriately conclude on the effects of pandemic. Having in mind that regular face-to-face check-ups were reduced, and people avoided visiting hospital, it might contribute to unsatisfying QoL scores in T1D patients. Our results would also be more accurate if data on participants’ psychological evaluation were avaliable. It is also unknown if controls in our study had other acute or chronic disease at the moment of data gathering, neither their COVID-19 status.

## 5. Conclusions

Results of our study suggest that, as it was expected, there is a significant difference in QoL between children with T1D and their controls. The lowest QoL scores were observed in the domain of school functioning, in rural area, as well among boys with T1D and in the central region. Children with T1D were deeply concerned regarding their health which emphasizes the need for psychological support. During COVID-19 pandemic, age has correlated with physical health in girls, which suggests that more attention should be paid to female students of higher grades. It is good to be prepared for some future public crisis with an anticipated strong impact on public health, by conducting QoL studies and monitoring quality of life of children with T1D during crisis, because that is the only way to have timely reactions and prevent consequences. Like that, results of our current study provide insights to prioritizing actions for children with diabetes care as well as for public healthcare planning.

## Figures and Tables

**Table 1:** Summary of participants’ socio-demographic characteristics.

	Cases (*n* = 87)	Controls (*n* = 248)	Total (*n* = 335)
Male %	62.1	60.9	61.2
Female %	37.9	39.1	38.8
Urban %	73.6	78.6	77.3
Rural %	26.4	21.4	22.7
Central Region %	48.3	48.0	48.1
North Region %	23.0	21.8	22.1
South Region %	28.7	30.2	29.9
Median of age in years (IQR)	12.41 (10.4–13.4)	12.1 (10.7–13.1)	12.2 (10.6–13.1)

**Table 2:** Medians (IQR) of scores by sociodemographic status and by case/control status.

		Physical Functioning	Emotional Functioning	Social Functioning	School Functioning	Psychosocial Health Summary	Physical Health Summary	Total Score
**T1D Status**	Case	90.6 (81.3–100)	80 (62.5–90)	100 (85–100)	75 (65–85)	81.7 (72.5–90)	90.6 (81.3–100)	85.9 (76.1–91.3)
	Control	93.7 (87.5–100)	85 (75–95)	100 (90–100)	90 (80–100)	90.8 (83.3–96.7)	93.8 (87.5–100)	91.3 (84.8–95.7)
	Test *	W = 9211 (*p* = 0.039)	W = 7904 (*p* < 0.001)	W = 9874.5 (*p* = 0.194)	W = 4711 (*p* < 0.001)	W = 5835 (*p* < 0.001)	W = 9307.5 (*p* = 0.0532)	W = 6653 (*p* < 0.001)
**Male**	Case	90.6 (81.3–100)	80 (65–90)	100 (90–100)	70 (60–85)	80.8 (73.3–90)	90.6 (81.3–100)	86.4 (76.1–91.3)
	Control	93.8 (87.5–100)	90 (80–100)	100 (90–100)	90 (85–100)	91.7 (85–96.7)	93.8 (87.5–100)	91.3 (86.4–96.7)
	Test *	W = 3642.5 (*p* = 0.238)	W = 2908.5 (*p* = 0.001)	W = 3886 (*p* = 0.575)	W = 1563 (*p* < 0.001)	W = 2015.5 (*p* < 0.001)	W = 3700.5 (*p* = 0.307)	W = 2411.5 (*p* < 0.001)
**Female**	Case	87.5 (81.3–96.9)	80 (60–90)	95 (85–100)	80 (65–85)	85 (71.7–90)	87.5 (81.3–96.9)	85.9 (75–91.3)
	Control	93.8 (87.5–100)	85 (70–95)	100 (90–100)	90 (80–100)	90 (81.7–96.7)	93.8 (87.5–100)	91.3 (82.6–95.7)
	Test *	W = 1267.5 (*p* = 0.071)	W = 1208 (*p* = 0.035)	W = 1376.5 (*p* = 0.183)	W = 860 (*p* < 0.001)	W = 973 (*p* < 0.001)	W = 1267.5 (*p* = 0.071)	W = 1034.5 (*p* = 0.002)
**Urban**	Case	87.5 (81.3–100)	80 (60–90)	100 (85–100)	77.5 (65–85)	80.8 (72.9–90)	87.5 (81.3–100)	85.3 (75.8–91.3)
	Control	93.8 (87.5–100)	85 (75–95)	100 (90–100)	90 (80–100)	90 (84.2–96.7)	93.8 (87.5–100)	91.3 (85.3–95.6)
	Test *	W = 5055 (*p* = 0.021)	W = 4550 (*p* = 0.001)	W = 5560.5 (*p* = 0.150)	W = 2752.5 (*p* < 0.001)	W = 3278.5 (*p* < 0.001)	W = 5135 (*p* = 0.031)	W = 3744 (*p* < 0.001)
**Rural**	Case	93.8 (84.4–100)	80 (70–90)	100 (92.5–100)	65 (60–82.5)	83.3 (73.3–88.3)	93.8 (84.4–100)	87 (79.3–90.8)
	Control	93.8 (87.5–100)	90 (80–100)	100 (90–100)	90 (80–100)	91.7 (83.3–96.7)	93.8 (87.5–100)	92.4 (82.6–97.8)
	Test *	W = 580.5 (*p* = 0.742)	W = 431.5 (*p* = 0.041)	W = 593.5 (*p* = 0.843)	W = 268.5 (*p* < 0.001)	W = 367 (*p* = 0.006)	W = 580.5 (*p* = 0.742)	W = 412.5 (*p* = 0.026)
**Central Region**	Case	87.5 (81.3–99.2)	77.5 (60–90)	100 (90–100)	70 (65–85)	80 (71.7–87.9)	87.5 (81.3–99.2)	82.6 (76.1–91.3)
	Control	93.8 (87.5–100)	85 (75–95)	100 (90–100)	90 (80–100)	90 (81.7–96.7)	93.8 (87.5–100)	91.3 (83.7–95.7)
	Test *	W = 2069 (*p* = 0.093)	W = 1758.5 (*p* = 0.004)	W = 2322.5 (*p* = 0.453)	W = 1175 (*p* < 0.001)	W = 1361.5 (*p* < 0.001)	W = 2063.5 (*p* = 0.089)	W = 1498 (*p* < 0.001)
**North Region**	Case	93.8 (87.3–100)	90 (68.8–90)	97.5 (85–100)	80 (63.8–86.3)	86.7 (76.7–90)	95.3 (87.5–100)	88.6 (82.3–91.8)
	Control	93.8 (87.5–96.9)	92.5 (80–100)	100 (95–100)	95 (85–100)	94.2 (86.7–96.7)	93.8 (87.5–95.9)	94 (86.4–97.6)
	Test *	W = 555.5 (*p* = 0.853)	W = 382 (*p* = 0.052)	W = 424.5 (*p* = 0.1)	W = 212.5 (*p* < 0.001)	W = 256.5 (*p* < 0.001)	W = 581 (*p* = 0.617)	W = 340 (*p* = 0.015)
**South Region**	Case	90.6 (81.3–100)	85 (60–90)	100 (85–100)	80 (65–85)	83.3 (73.3–90)	90.6 (81.3–100)	85.9 (75–91.3)
	Control	93.8 (87.5–100)	85 (75–100)	95 (90–100)	90 (85–100)	90 (85–95)	93.8 (87.5–100)	91.3 (87–95.1)
	Test *	W = 720 (*p* = 0.078)	W = 728 (*p* = 0.093)	W = 924.5 (*p* = 0.915)	W = 372 (*p* < 0.001)	W = 536 (*p* = 0.001)	W = 720 (*p* = 0.078)	W = 579 (*p* = 0.004)

* Mann-Whitney U test.

**Table 3:** Correlation between Physical functioning score and age by gender and household type.

	*ρ* *	*p*-Value
Total	−0.14	0.009
Male	0.04	0.576
Female	−0.37	*p* < 0.001
Urban	−0.13	0.031
Rural	−0.16	0.177

* Spearman’s rank correlation coefficient.

**Table 4:** Patients’ reports on frequency of different diabetes-related problems during the month before the research interview (*n* = 87).

	Percentage				
	Never	Almost Never	Sometimes	Often	Almost Always
I feel hungry	12.8	10.5	48.8	19.8	8.1
I feel thirsty	23.2	29.1	41.9	5.8	0
I have to go to the bathroom too often	45.3	32.6	17.4	3.5	1.2
I have tummy aches	39.6	29.1	26.7	2.3	2.3
I have headaches	54.7	26.7	14	3.4	1.2
I feel like I need to throw up	73.3	17.4	7	2.3	0
I go “low”	4.7	9.3	67.4	18.6	0
I go “high”	4.7	9.3	67.4	18.6	0
I feel tired	32.5	29.1	29.1	7	2.3
I get shaky	44.2	19.8	20.8	14	1.2
I get sweaty	52.3	23.3	22.1	0	2.3
I feel dizzy	62.8	23.3	10.4	2.3	1.2
I feel weak	34.9	29.1	25.6	8.1	2.3
I have trouble sleeping	70.9	18.6	4.7	2.3	3.5
I get cranky or grumpy	27.9	18.6	40.7	8.1	4.7

## Data Availability

Data supporting reported results are available upon request. PedsQL™—Pediatric Quality of Life Inventory™ questionnaires, contact information and permission to use from Mapi Research Trust, Lyon, France, https://eprovide.mapi-trust.org, (accessed on 21 August 2022).
